# Musculoskeletal disorders among teachers: a systematic review and meta-analysis

**DOI:** 10.3389/fpubh.2024.1399552

**Published:** 2024-10-04

**Authors:** Somayeh Tahernejad, Anahita Hejazi, Ehsan Rezaei, Faezeh Makki, Ali Sahebi, Zahra Zangiabadi

**Affiliations:** ^1^Health in Disasters and Emergencies Research Center, Institute for Futures Studies in Health, Kerman University of Medical Sciences, Kerman, Iran; ^2^Non-Communicable Diseases Research Center, Ilam University of Medical Sciences, Ilam, Iran; ^3^Department of Medical Emergencies and Health in Disasters and Emergencies, Ilam University of Medical Sciences, Ilam, Iran

**Keywords:** musculoskeletal disorders, teachers, risk factors, prevention, ergonomics

## Abstract

**Introduction:**

As a result of the demands of their profession, teachers encounter a range of ergonomic risk factors and are highly susceptible to developing musculoskeletal disorders (MSDs). Accordingly, this systematic review and meta-analysis was carried out to examine the frequency of MSDs among teachers.

**Materials and methods:**

The present research followed the preferred reporting items for systematic reviews and meta-analyses (PRISMA) guidelines and its protocol was registered in international prospective register of systematic review (PROSPERO) under the code CRD42024509263. To conduct the searches, various databases such as PubMed, Scopus, Web of Science, Science Direct, SID, ISC, and Google Scholar were utilized, and the search period was until February 7th, 2024 without time restriction. A random effects model was employed for meta-analysis, and I^2^ index was utilized to assess heterogeneity among the studies. Data analysis was carried out using STATA (version 14).

**Results:**

After an initial search across the mentioned databases, a total of 2,047 articles were identified. Following screening, study selection, and quality evaluation, 44 studies were ultimately chosen for meta-analysis, involving 15,972 teachers. The results of the meta-analysis revealed that the overall prevalence of MSDs among teachers is 68% (95% CI: 61–75, I^2^ = 99.2%, *p* < 0.001). Furthermore, the prevalence rates of MSDs in different body regions, such as the neck (47%), lower back (47%), shoulder (44%), upper back (37%), knee (35%), ankle (30%), wrist (27%), hip (22%), and elbow (13%), were reported.

**Conclusion:**

The overall prevalence of MSDs among teachers is relatively high. Neck and lower back pain are more common among them compared to other body regions. It is recommended that periodic occupational medicine examinations, training, and the implementation of ergonomic interventions for this occupational group focus on assessing the risk factors for MSDs, especially in the neck and lower back regions.

**Systematic review registration:**

https://www.crd.york.ac.uk/prospero/display_record.php?RecordID=509263, identifier CRD42024509263.

## 1 Introduction

Work-related musculoskeletal disorders (WMSDs) are presently prevalent and expensive occupational health issues in both developing and developed nations (1, 2). Globally, WMSDs are a significant concern leading to substantial direct and indirect costs for employees, employers, and governments (3). To date, numerous studies have reported a wide range of risk factors associated with MSDs, which have been classified into four general categories: mental risk factors (4), individual and personality risk factors (5), biodynamic risk factors (6), and hidden risk factors (7).

Teachers are also among the professionals who encounter diverse ergonomic risk factors (8). Teachers’ responsibilities involve repetitive duties, fixed postures, and occasionally extended work hours (9). Apart from teaching (10), teachers also engage in evaluating students’ schoolwork or homework (11). Sometimes teachers have to lift heavy items, such as books, educational materials, and laptops. Additionally, due to the demands of their profession, teachers may maintain a posture for longer periods than recommended ergonomically, primarily because of the high mental workload, thereby exposing themselves to the challenges associated with prolonged static work. At times, teachers adopt awkward postures, such as tilting the neck backward while writing on the board or leaning the neck forward during tasks like reviewing lessons, assessing assignments, marking exams, using computers, and handling school administrative duties. Moreover, regularly writing on the board with hands positioned above shoulder level, standing for prolonged periods in improper positions during teaching, sitting inappropriately, and frequently twisting or bending from the side of the board towards the students and back can result in strains. These behaviors may adversely impact the musculoskeletal system of teachers (9, 12–21).

Teachers may find themselves pushing their physical, cognitive, and emotional limits to meet educational objectives. Insufficient time for recuperation can trigger or worsen pain symptoms, potentially leading to stress that adversely affects both physical and mental well-being of teachers and consequently impacts their professional performance (22). Besides physical and psychological aspects, individual factors such as the gender and age of teachers are also associated with MSDs (23). Given the crucial role that teachers play in enhancing and ensuring the quality of the education process, the well-being of their musculoskeletal system holds significant importance.

Research indicates a growing apprehension regarding the risk of WMSDs in the realm of education, with WMSDs generally being more prevalent among school teachers compared to other professional groups (13). Some studies have indicated WMSDs as a significant and costly occupational health issue, resulting in a decline in teachers’ quality of life (1), and sometimes, these disorders may necessitate teachers to take extended sick leave (8). Furthermore, WMSDs have been identified as a contributing factor to the premature retirement of teachers (24, 25). Another study indicated that the occurrence of MSDs among teachers could impact their daily tasks, such as work responsibilities, potentially resulting in higher rates of absenteeism (22).

Given the significance of MSDs as a prevalent and crucial occupational health issue in teaching profession (10), it appears essential to carry out thorough and credible epidemiological studies in the realm of MSDs among teachers. The prevalence of MSDs in various body parts can provide important information regarding some ergonomic risk factors associated with each occupation (26). Surveys show that so far, several studies have evaluated the prevalence of MSDs among teachers, but according to the results of our surveys, no comprehensive study was found that studied the overall prevalence and types of MSDs among this occupational group. Due to the importance of WMSDs, several systematic reviews and meta-analyses have been conducted to estimate the overall prevalence of MSDs among employees of different occupational groups, for example dentists (27), orthopedic surgeons (28), sanitary workers (29), firefighters (30), nurses (31, 32), operating room personnel (33), and physiotherapists (34, 35). In some studies, the prevalence of occupational low back pain among occupational groups has been specifically investigated (36, 37). The findings from each of these studies can be useful to assess the work ability index or develop strategies to enhance this index across the respective occupations.

Based on the aforementioned materials, understanding the overall prevalence of MSDs and the prevalence of these disorders in various body parts to reduce them is necessary for each occupational group. In fact, this knowledge is one of the most important measures for designing effective ergonomic interventions to maintain the health of the workforce. Hence, considering the significance of the topic, the present study aimed to provide more comprehensive information regarding the investigation of MSDs among teachers through a systematic review and meta-analysis. The findings of this study can serve as an important source of information for occupational health managers to develop more effective ergonomic interventions and ergonomic training programs to prevent teachers from suffering from MSDs.

## 2 Methods

The systematic review and meta-analysis were carried out following the guidelines of the Preferred Reporting Items for Systematic Reviews and Meta-Analyses (PRISMA) (38). The study protocol is currently registered in the International Prospective Register of Systematic Reviews (PROSPERO) under the code CRD42024509263. Various phases of the study, such as the search strategy, screening, study selection, quality assessment, and data extraction, were conducted following the PRISMA protocol. The final three stages, i.e., study selection, quality evaluation, and data extraction, were carried out independently by two researchers, with any disagreements resolved through group discussion.

### 2.1 Sources of information and search strategy

In this study, information sources include PubMed, Scopus, Web of Science, Science Direct, SID, ISC, Google Scholar, conference and congress articles, as well as the bibliography of selected articles and systematic review studies, which were utilized for searching and extracting relevant studies. To extract valid keywords, we recognized MeSH terms (formal words or phrases selected to denote specific biomedical concepts (39)), utilized the keywords from pertinent articles, and sought advice from scientific experts. The search strategies for all databases were developed using the following appropriate keywords:

“Teachers*,” “High school teachers*,” “Preschool teachers*,” “Secondary school teachers*,” “School teacher*,” “Primary school teachers*,” “Musculoskeletal disorder*,” “Musculoskeletal disease*,” “Work related Musculoskeletal disorder*,” “Muscle strain*,” “Musculoskeletal symptom*,” “Musculoskeletal complaint*,” “WRMSDs,” “Muscle problem*,” “Dysfunction*,” “Neck pain*,” “Musculoskeletal problem*,” “MSDs,” “Musculoskeletal pain*,” “Arthritis joint*,” “Arthritis bone*,” “Shoulder pain*,” “Elbow pain*,” “Back pain*,” and “Hand pain*.”

Additionally, several operators and search fields were utilized to combine keywords. The search period was until February 7th, 2024 without time constraints. Table 1 presents the search strategy for all database types.

**Table 1:** Search strategy in various databases.

Database	Search strategy
PubMed	((“Teachers*” OR “High school teachers*” OR “Preschool teachers*” OR “Secondary school teachers *” OR “School teacher*” OR “Primary school teachers *”) AND (“Musculoskeletal disorder*” OR “Musculoskeletal disease*” OR “Work related Musculoskeletal disorder*” OR “Muscle strain*” OR “Musculoskeletal symptom*” OR “Musculoskeletal complaint*” OR “WRMSDs” OR “Muscle problem*” OR “Dysfunction*” OR “Neck pain *” OR “Musculoskeletal problem*” OR “MSDs” OR “Musculoskeletal pain*” OR “Arthritis joint*” OR “Arthritis bone*” OR “Shoulder pain*” OR “Elbow pain*” OR “Back pain*” OR “Hand pain*”))
Scopus	(((TITLE-ABS-KEY(“Teachers*”) OR TITLE-ABS-KEY(“High school teachers*”) OR TITLE-ABS-KEY(“Preschool teachers*”) OR TITLE-ABS-KEY(“Secondary school teachers*”) OR TITLE-ABS-KEY(“School teacher*”) OR TITLE-ABS-KEY(“Primary school teachers*”)) AND (TITLE-ABS-KEY(“Musculoskeletal disorder*”) OR TITLE-ABS-KEY(“Musculoskeletal disease*”) OR TITLE-ABS-KEY(“Work related Musculoskeletal disorder*”) OR TITLE-ABS-KEY(“Muscle strain*”) OR TITLE-ABS-KEY(“Musculoskeletal symptom*”) OR TITLE-ABS-KEY(“Musculoskeletal complaint*”) OR TITLE-ABS-KEY(“WRMSDs”) OR TITLE-ABS-KEY(“Muscle problem*”) OR TITLE-ABS-KEY(“Dysfunction*”) OR TITLE-ABS-KEY(“Neck pain*”) OR TITLE-ABS-KEY(“Musculoskeletal problem*”) OR TITLE-ABS-KEY(“MSDs”) OR TITLE-ABS-KEY(“Musculoskeletal pain*”) OR TITLE-ABS-KEY(“Arthritis joint*”) OR TITLE-ABS-KEY(“Arthritis bone*”) OR TITLE-ABS-KEY(“Shoulder pain*”) OR TITLE-ABS-KEY(“Elbow pain*”) OR TITLE-ABS-KEY(“Back pain*”) OR TITLE-ABS-KEY(“Hand pain*”))))
Web Of Science (WOS)	(((TS = (“Teachers *”) OR TS = (“High school teachers *”) OR TS = (“Preschool teachers *”) OR TS = (“Secondary school teachers *”) OR TS = (“School teacher *”) OR TS = (“Primary school teachers*”)) AND (TS = (“Musculoskeletal disorder *”) OR TS = (“Musculoskeletal disease”) OR TS = (“Work related Musculoskeletal disorder *”) OR TS = (“Muscle strain”) OR TS = (“Musculoskeletal symptom*”) OR TS = (“Musculoskeletal complaint *”) OR TS = (“WRMSDs”) OR TS = (“Muscle problem *”) OR TS = (“Dysfunction *”) OR TS = (“Neck pain *”) OR TS = (“Musculoskeletal problem *”)OR TS = (“MSDs *”) OR TS = (“Musculoskeletal pain *”) OR TS = (“Arthritis joint *”) OR TS = (“Arthritis bone *”) OR TS = (“Shoulder pain *”) OR TS = (“Elbow pain *”) OR TS = (“Back pain *”) OR TS = (“Hand pain *”))))

### 2.2 Inclusion criteria

For this research, studies with focus on MSDs among teachers were included.

### 2.3 Exclusion criteria

Review studies, case reports, intervention studies, non-English papers, letters to the editor, and reports on the prevalence of musculoskeletal injuries resulting from accidents were not included in the study.

### 2.4 Study selection process

Initially, all identified articles were imported into EndNote X7 for organization. Following the removal of duplicates, the titles and abstracts of the remaining articles were reviewed based on specific criteria. Subsequently, potentially relevant articles were pinpointed, and two researchers separately examined the full text of these articles. Ultimately, articles meeting the eligibility criteria were chosen.

### 2.5 Quality assessment and data extraction

During this phase, two researchers independently assessed the quality of the chosen studies utilizing the Appraisal tool for Cross-Sectional Studies (AXIS) (40). The scoring system of this tool assigns values from 0 to 20. For this study, articles scoring 1^2^ or higher were included in meta-analysis (30). It is important to mention that no study was omitted from this research. In the following stage, two researchers separately gathered the data. In this phase, details such as the author’s name, sample size, gender distribution, average age of participants, prevalence of MSDs and their types, and assessment tools of each study were obtained and recorded in a pre-prepared checklist.

### 2.6 Statistical analysis

To determine the variance of each study, the binomial distribution was utilized. Moreover, a weighted mean was used to combine the prevalence of MSDs across various studies. The weighting of each study was determined by its inverse variance. A simple random effects model was employed for conducting the meta-analysis.

The I^2^ index was utilized to assess the level of heterogeneity among the studies. Heterogeneity was categorized into various ranges: less than 25%, 25–50%, 50–75%, and above 75%, indicating no heterogeneity, medium heterogeneity, high heterogeneity, and very high heterogeneity, respectively (41). Begg’s test was employed to examine publication bias. Ultimately, the study data were analyzed using STATA software (version 14).

## 3 Result

### 3.1 Systematic review results

**Figure 1:**
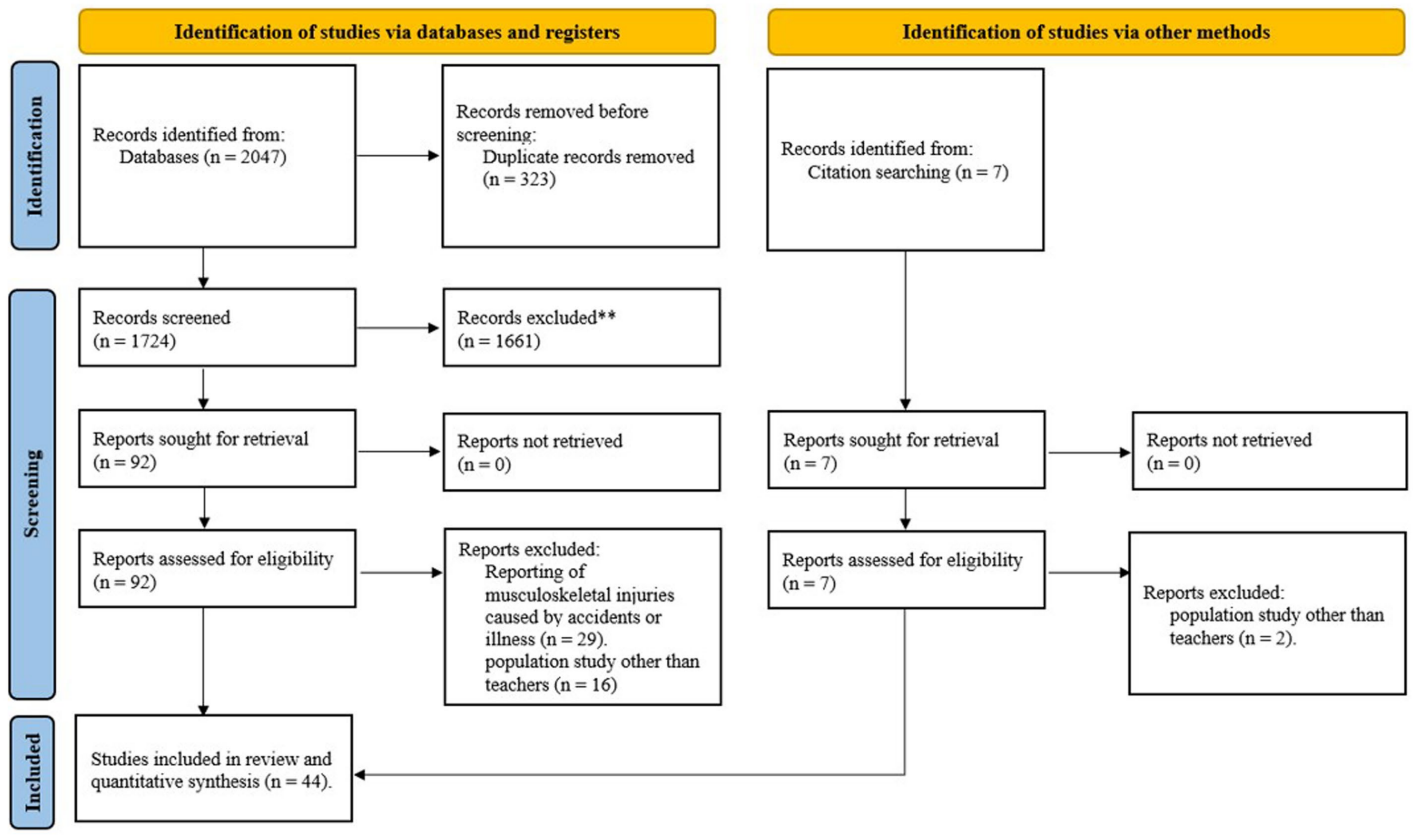
Flowchart of study selection based on PRISMA.

At first, a total of 2,047 articles were found in the initial database search. Subsequently, 1,724 studies were retained after eliminating duplicates and entered the screening phase. Following this process, 92 articles were selected for further full-text examination. Then, a total of 44 studies were eventually chosen for qualitative assessment. These 44 studies entered the meta-analysis phase (Figure 1). This study examined the prevalence of MSDs among 15,972 teachers. Among the 44 studies, 37 reported the overall prevalence of MSDs, involving 14,069 teachers. A summary of the characteristics of the selected studies is provided in Table 2.

**Table 2:** The specifications of studies included in the meta-analysis.

First author/Year	Country	Sample size	Total prevalence of MSDs	Prevalence of MSD types	Tools^a^
Sankar (2024) (50)	India	400 (female: 315 (78.75%)/ male: 85 (21.25%))	69.8%	Neck: 25.5%	NMQ
				Shoulder: 17.8%	
				Elbows: 6.0%	
				Wrist/hands: 11%	
				Upper back: 4.8%	
				Lower back: 9.8%	
				Hips/thigh: 24.3%	
				Knee: 23.3%	
				Ankle/feet: 14%	
De Souza (2023) (22)	Brazil	246 (female: 187/male: 59)	NR	Lower back: 40.7%	NMQ
				Elbow: 11%	
				Shoulder: 37.8%	
				Neck: 40.2%	
				Knee: 24.8%	
				Hip/thigh: 16.7%	
				Ankle/foot: 26%	
				Upper back: 32.1%	
				Wrist/hand: 29.7%	
Da Cruz Teles (2023) (51)	Brazil	326	76.1%	NR	NMQ
Grabara (2023) (52)	Poland	254 (female: 203/male: 51)	NR	Lower back: 57.1%	NMQ
				Shoulder: 26.4%	
				Knee: 37.4%	
				Neck: 52.8%	
				Upper back: 38.6%	
				Ankles/foot: 23.6%	
				Wrist/hand: 22.8%	
				Hip/thigh:18.9%	
				Elbow: 11%	
Ramírez-García (2023) (53)	Ecuador	134 (female: 86/male: 48)	67%	Neck: 69%	NMQ
				Shoulder: 46%	
				Back/lumbar: 49%	
				Elbow: 16%	
				Wrist/hand: 33%	
AlMaghlouth (2022) (54)	Saudi Arabia	404 (female: 211 (52.2%)/ male: 193 (47.8%))	41.1%	Neck: 62.1%	NMQ
				Shoulders: 69.8%	
				Elbows: 33.7%	
				Hands: 48.3%	
				Back: 80.2%	
				Hips/thighs: 45%	
				Knees: 62.9%	
				Ankles: 55%	
Althomali (2022) (55)	Saudi Arabia	251 (male:145/ Female:106)	93.63%	Lower back: 72.91%	NMQ
				Neck: 49.8%	
				Shoulders: 66.93%	
				Elbows: 17.93%	
				Wrist/hands: 39.44%	
				Upper back: 42.63%	
				Hips/thighs/buttocks: 43.03%	
				Knees: 46.61%	
				Ankles/feet: 37.85%	
Alajmi (2022) (56)	Saudi Arabia	372	86%	Right shoulder: 43.5%	NMQ
				Neck: 62.9%	
				Left shoulder: 51.6%	
				Upper back: 48.9%	
				Right humerus: 21.5%	
				Left humerus: 25.3%	
				Lower back: 53.8%	
				Right arm: 23.7%	
				Left arm: 23.7%	
				Right wrist: 24.7%	
				Left wrist: 18.8%	
				Hip: 39.2%	
				Right thigh: 26.3%	
				Left thigh: 24.7%	
				Right knee: 37.1%	
				Left knee: 40.9%	
				Right leg: 26.9%	
				Left leg: 25.8%	
				Right foot: 32.8%	
				Left foot: 36%11	
Celikkalp (2022) (57)	Turkey	416	64.9%	Neck: 55.5%	CMDQ
				Lower back: 47.6%	
				Back: 53.4%	
				Elbow: 6.5%	
Gabani (2022) (58)	Brazil	958	43%	Lower back: 11%	Interview
				Upper limb: 14%	
				Lower limb: 13%	
De Souza (2022) (59)	Brazil	245	NR	Neck: 19.6%	NMQ
				Shoulders: 18.0%	
				Upper back: 12.3%	
				Elbows: 3.7%	
				Wrists/hands: 14.3%	
				Lower back: 19.3%	
				Hip/thighs: 8.6%	
				Ankles/feet: 13.1%	
				Knees: 12.7%	
Fahmy (2022) (1)	Egypt	310 (female: 205/ male: 105)	66.77%	Neck: 56.1%	NMQ
				Shoulder: 53.2%	
				Lower back: 53.2%	
				One or both knee: 50.6%	
				Wrists/hands: 39%	
				Upper back: 33.2%	
				One or both ankles/feet: 28.7%	
				Elbow: 15.2%	
Matias (2022) (60)	Brazil	60 (female: 38/male: 22)	75%	Cervical spine: 45%	NMQ
				Lumbar spine: 68.3%	
				Thoracic spine: 41.7%	
				Wrists/hands: 41.7%	
				Shoulders: 23.3%	
				Arms: 15.0%	
				Forearms: 6.7%	
				Hip: 23.3%	
				Thighs: 15%	
				Knees: 26.7%	
				Legs: 8.3%	
				Ankles/feet: 23.3%	
Moreto (2022) (61)	Perú	91 (male: 46/ Female: 45)	58.2%	Shoulder: 51.6%	NMQ
				Neck: 52.7%	
				Wrists/hands: 52.7%	
				Elbow/forearms: 48.4%	
				Lumbar thoracic: 51.6%	
Mekoulou Ndongo (2022) (62)	Cameroon	179	84.3%	Lower back: 43%	NMQ
				Shoulder: 35%	
				Neck: 33.5%	
				Knee:12.4%	
				Hips/thigh:11.2%	
				Wrist/hand:25.7%	
				Ankles/feet:1%	
				Elbow:4.5%	
				Upper back:25.1%	
Althomali (2021) (63)	Saudi Arabia	251 (male: 57.8%/ females: 42.2%)	87.3%	Neck: 36.25%	NMQ
				Shoulders: 53.4%	
				Elbows: 16.3%	
				Wrist/hands: 30.3%	
				Upper back: 33.5%	
				Lower back: 62.55%	
				Hips/thighs/buttocks: 37.05%	
				Knees: 41.04%	
				Ankles/feet: 31.5%	
Arshad (2021) (64)	Pakistan	289	82.7%	Neck: 50.2%	NMQ
				Shoulders: 44.3%	
				Elbows: 26.3%	
				Wrist/hands: 30.8%	
				Upper back: 43.3%	
				Lower back: 60.2%	
				One or both hips/thighs: 32.9%	
				One or both knees: 37.4%	
				One or both ankles: 48.4%	
Abdel-Salam (2021) (65)	Saudi Arabia	254 (female)	68.50%	Low back: 68.4%	Self-administered questionnaire
				knee: 58.6%	
				Shoulder: 47.7%	
				Neck: 45.4%	
				Elbow: 23.6%	
				Wrist: 14.4%	
Aldukhayel (2021) (66)	Saudi Arabia	503	91%	Back: 74.4%	NMQ
				Elbow: 13.1%	
				Shoulder: 57.5%	
				Neck: 48.5%	
				Knee: 10.1%	
				Wrist/hand: 22.1%	
Vega-Fernandez (2021) (44)	Chile	153	71.2%	Neck: 44.4%	NMQ
				Shoulders: 32.7%	
				Neck/shoulders: 53.6%	
				Elbows: 13.7%	
				Wrist/hand: 26.8%	
				Any upper limb: 61.4%	
				Upper back: 32%	
				Low back: 43.1%	
				Any back: 51%	
				Hips/thigh: 24.2%	
				Knee: 34%	
				Ankles/feet: 24.8%	
				Any lower limb: 49%	
Khalid (2021) (67)	Pakistan	921 (male: 686 (74.8%)/ female: 232 (25.2%))	70.8%	Neck: 17%	Self-administered questionnaire
				Upper extremity: 28%	
				Lower extremity: 9.1%	
				Lower back: 16.6%	
Souza (2021) (68)	Brazil	304	24.3%	Back: 15.5%	NMQ
				Upper limbs: 16.1%	
				Lower limbs: 12.5%	
Alharbi (2020) (69)	Saudi Arabia	400 (male)	62.5%	Shoulder: 47.9%	Self-administered questionnaire
				Neck: 41.3%	
				Lower back: 59.2%	
				Elbow: 11.7%	
				Wrists/hand: 8.8%	
				Lower limbs: 43.3%	
Amit (2020) (70)	Philippines	200	74.5%	Neck: 47.5%	Musculoskeletal burdens work hazards (H-9-2016) questionnaire
				Lower back/waist: 56.0%	
				Shoulder: 45.0%	
				Arm/elbow: 33.0%	
				Hand/wrist: 44.0%	
				Leg/foot: 56.5%	
Alias (2020) (71)	Malaysia	212	40.1%	Neck: 22.6%	NMQ
				Shoulder: 22.2%	
				Upper back: 26.4%	
				Lower back: 25.0%	
				Elbow: 10.4%	
				Hands: 9.9%	
				Arm: 11.3%	
				Knee: 28.8%	
				Thigh: 18.4%	
				Feet: 32.5%	
Arvidsson (2020) (72)	Sweden	246 (female)	21%	Neck: 40%	NMQ
				Shoulder: 34%	
				Feet:11%	
				Hand: 16%	
				Lower back: 38%	
Chand (2020) (73)	Pakistan	255 (female: 164 (64.3%)/ male: 91 (35.7%))	88.9%	Neck: 48.5%	Self-administered questionnaire
				Shoulder: 46.6%	
				Upper back: 25.6%	
				Lower back: 45.4	
				Elbow: 4.6%	
				Wrist/hand: 12.2%	
				Hip:9.5%	
				Leg: 23.3%	
				Knee: 21.8%	
				Ankle: 15.3%	
De Souza (2020) (47)	Brazil	224	NR	Neck: 19.6%	NMQ
				Thoracic: 33.9%	
				Shoulder: 18.8%	
				Elbow: 11. 11%	
				Wrist: 15.1%	
				Low back: 20.1%	
				Hip/thigh: 9.4%	
				Knee: 13.8%	
				Ankle/foot: 26.8%	
Kraemer (2020) (74)	Brazil	25 (male: 18 (72%)/female: 7 (28%))	100%	Neck: 56%	NMQ
				Shoulder: 48%	
				Lower back: 60%	
				Upper back: 40%	
				Wrists/hands: 32%	
				Elbow: 8%	
Ng (2019) (75)	Malaysia	367 (female: 318 (86.6%)/male: 49 (13.4%))	80.1%	Neck: 75.5%	CMDQ
				Shoulder: 80.1%	
				Upper back: 56.4%	
				Upper arm: 91.3%	
				Lower back: 59.9%	
				Forearm: 89.6%	
				Wrist: 93.2%	
				Hip/buttocks: 40.9%	
				Thigh: 91.8%	
				Knee: 88%	
				Lower leg: 90.5%	
				Foot: 87.7%	
Converso (2018) (76)	Italy	429	55.5%	Lower back: 70. 6%	NMQ
				Upper back: 84%	
				Neck: 75.6%	
				Shoulder: 56.3%	
				Hips/thigh: 49.6%	
				Knee: 49.6%	
				Wrist/hand: 38.7%	
				Ankles/feet: 16.8%	
				Elbow: 16%	
Ojukwu (2018) (77)	Nigeria	352 (female: 260/ male: 92)	70.2%	Shoulder: 62.3%	NMQ
				Neck: 57.9%	
Vaghela (2018) (78)	India	314	71.95%	Neck: 20.7%	NMQ
				Shoulder: 33.12%	
				Elbow: 4.3%	
				Wrist/hand: 15.75%	
				Upper back: 29.97%	
				Lower back: 49.92%	
				Hip/thigh: 7.01%	
				Knee: 33.73%	
				Ankle/foot: 25.41%	
Solis-Soto (2017) (79)	Bolivia	517	86%	Neck: 47.2%	NMQ
				Upper back: 35.8%	
				Hips/thighs: 31.9%	
				Lower back: 33.1%	
				Shoulder: 34.6%	
				Upper extremities: 46.7%	
				Upper extremities back: 63.7%	
				Ankles/feet: 30.4%	
				Wrist/Hands: 25.7%	
				Knees: 37.5%	
Zamri (2017) (80)	Malaysia	1,482	48%	Neck: 60.1%	NMQ
				Shoulder: 60.1%	
				Low back: 48%	
Arvidsson (2016) (81)	Sweden	375 (female)	NR	Lower back: 36%	NMQ
				Shoulder: 38%	
				Neck: 44%	
				Foot: 12%	
				Hand: 17%	
Ceballos (2015) (82)	Brazil	525 (female: 452/male: 73)	73.5%	Lower back: 18.3%	Self-administrated questionnaire
				Shoulder: 31.6%	
				Neck: 27.2%	
				Wrists/hands: 17.9%	
				Knee: 18.1%	
				Ankles/feet: 24%	
				Hip/thigh: 11%	
				Elbow: 4.4%	
				Upper back: 27.8%	
Karakaya (2015) (83)	Turkey	104	36%	Lower back: 38%	NMQ
				Neck: 39%	
				Shoulders: 28%	
				Elbows: 9%	
				Wrists/hands: 16%	
				Hips/thighs: 12%	
				Knees: 22%	
				Ankles/feet: 33%	
				Upper back: 32%	
Shuai (2014) (13)	China	328	NR	Shoulder: 52.29%	NMQ
				Neck: 63.43%	
				Lower back: 47.14%	
				Wrist/hand: 25.14%	
				Ankles/foot: 21.14%	
				Knee: 26.57%	
				Hip/thigh: 16.57%	
				Elbow: 13.14%	
				Upper back: 37.71%	
Cheng (2013) (84)	Taiwan	323	94%	Shoulder: 76.2%	NMQ
				Lower back:74.6%	
				Neck: 65.9%	
Darwish (2013) (20)	Saudi Arabia	240	79.17%	Lower back: 63.8%	NMQ
				Shoulder: 45.4%	
				Neck: 42.1%	
				Leg: 40.0%	
				Wrist: 16.2%	
				Elbow: 10.0%	
Mohammadi (2013) (85)	Iran	231 (male: 141/female: 90)	NR	Neck: 72.7%	NMQ
				Shoulder: 51.3%	
				Upper back: 62.6%	
				Lower back: 44.8%	
				Knee: 59.3%	
Durmus (2012) (18)	Turkey	602 (male: 312/female: 290)	60.3%	Neck: 47.9%	Self-administrated questionnaire
				Knee: 30.9%	
				Lower back: 74.9%	
				Shoulder: 55.9%	
				Elbow: 11%	
				Back: 42.7%	
				Ankle/foot: 29.5%	
				Wrist/hand: 23.4%	
				Hip/thigh: 15.4%	
Korkmaz (2011) (16)	Turkey	900	51.4%	Neck: 42.5%	Self-administered questionnaire
				Upper back: 36.9%	
				Lower back: 43.8%	
				Shoulder: 28.7%	
				Elbow: 8.0%	
				Wrist: 13.4%	
				Hip: 8.4%	
				Knee: 32.0%	
				Ankle: 21.8%	
				Neck: 42.5%	

a NMQ, Nordic Musculoskeletal Questionnaire; CMDQ, Cornell Musculoskeletal Discomfort Questionnaires.

NR, not reported.

### 3.2 Characteristics of included studies

**Figure 2:**
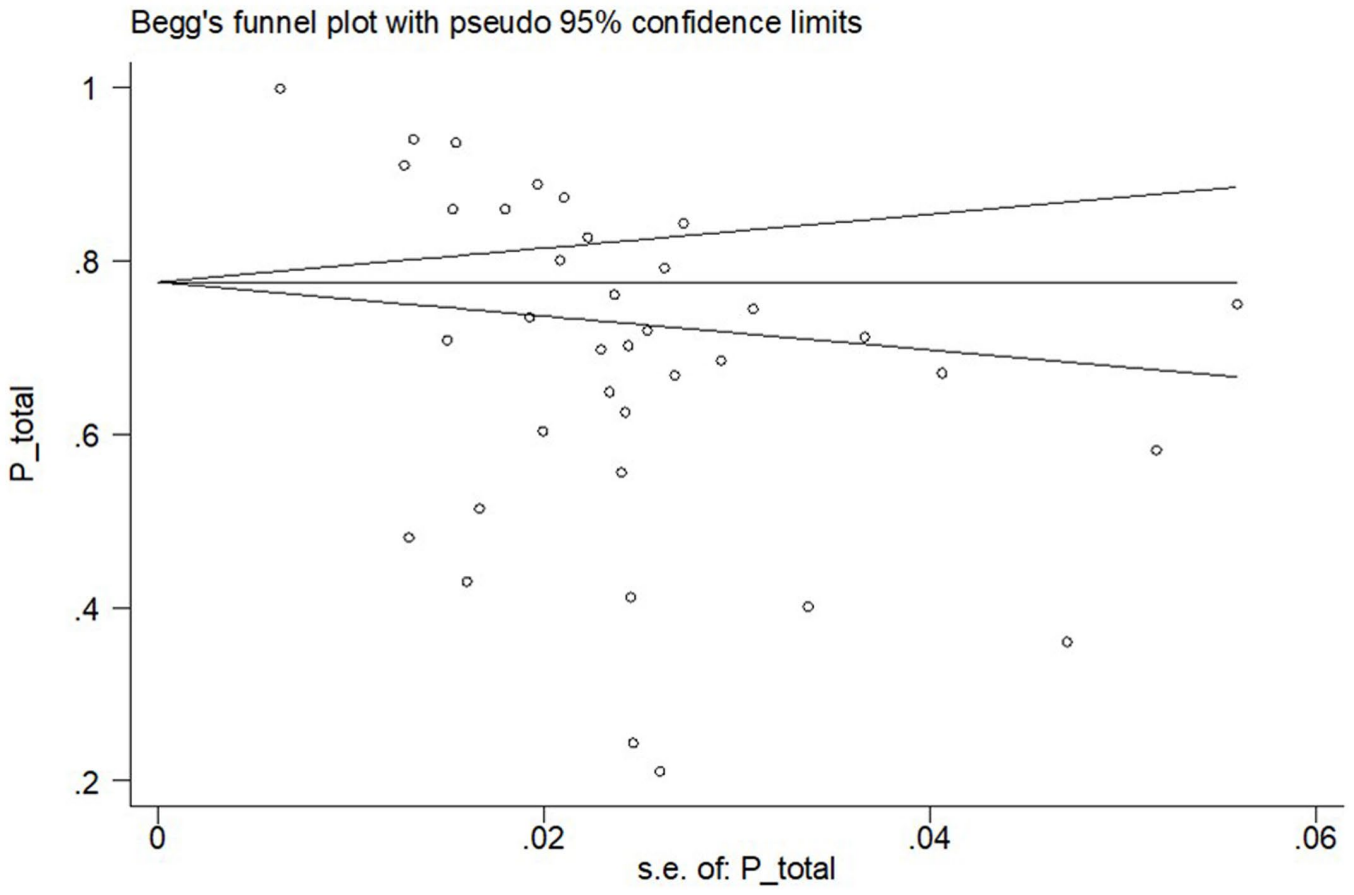
The overall prevalence of MSDs among teachers and the 95% confidence interval for each of the reviewed studies and all studies.

According to the findings of the meta-analysis, 68% (95% CI: 61–75, I^2^ = 99.2%, *p* < 0.001) of teachers experienced MSD symptoms in at least one region of their body. The level of heterogeneity among the reviewed studies, as indicated by the I^2^ index in this study, was very high (Figure 2). The findings of the subgroup analysis on the prevalence of MSDs across different body regions are presented in Table 3. As per the results, the lower back and neck had the highest prevalence, respectively, at 47% (95% CI: 40–55, I^2^ = 99.1%, *p* < 0.001), and 47% (95% CI: 41–52, I^2^ = 98%, *p* < 0.001), while the elbow had the lowest prevalence of MSDs at 13% (95% CI: 11–16, I^2^ = 93.5%, *p* < 0.001). Additionally, I^2^ values calculated for all investigated regions were notably high.

**Table 3:** Meta-analysis results for different body regions.

MSDs	Number of studies	Sample size	Prevalence of MSDs	95% CI	I^2^	Begg’s test	Egger’s test
Lower back	41	15,112	47%	40–55%	99.1%	*p* = 0.946	*p* = 0.003*
Neck	41	14,710	47%	41–52%	98%	*p* = 0.982	*p* = 0.306
Shoulder	38	12,675	44%	39–50%	97.9%	*p* = 0.555	*p* = 0.493
Wrist	34	10,287	27%	19–35%	99.1%	*p* < 0.001*	*p* = 0.963
Elbow	31	9,715	13%	11–16%	93.5%	*p* < 0.001*	*p* < 0.001*
Upper back	29	10,216	37%	30–45%	98.7%	*p* = 0.574	*p* = 0.071
Knee	27	8,807	35%	27–43%	98.8%	*p* = 0.108	*p* = 0.445
Ankle	27	8,634	30%	21–38%	99.1%	*p* = 0.015*	*p* = 0.006*
Hip	25	8,437	22%	18–27%	97.4%	*p* = 0.001*	*p* = 0.001*

CI, confidence interval; I^2^, I squared. The asterisk (*) indicates a significant difference (*p* < 0.05).

### 3.3 Risk of bias assessment

**Figure 3:**
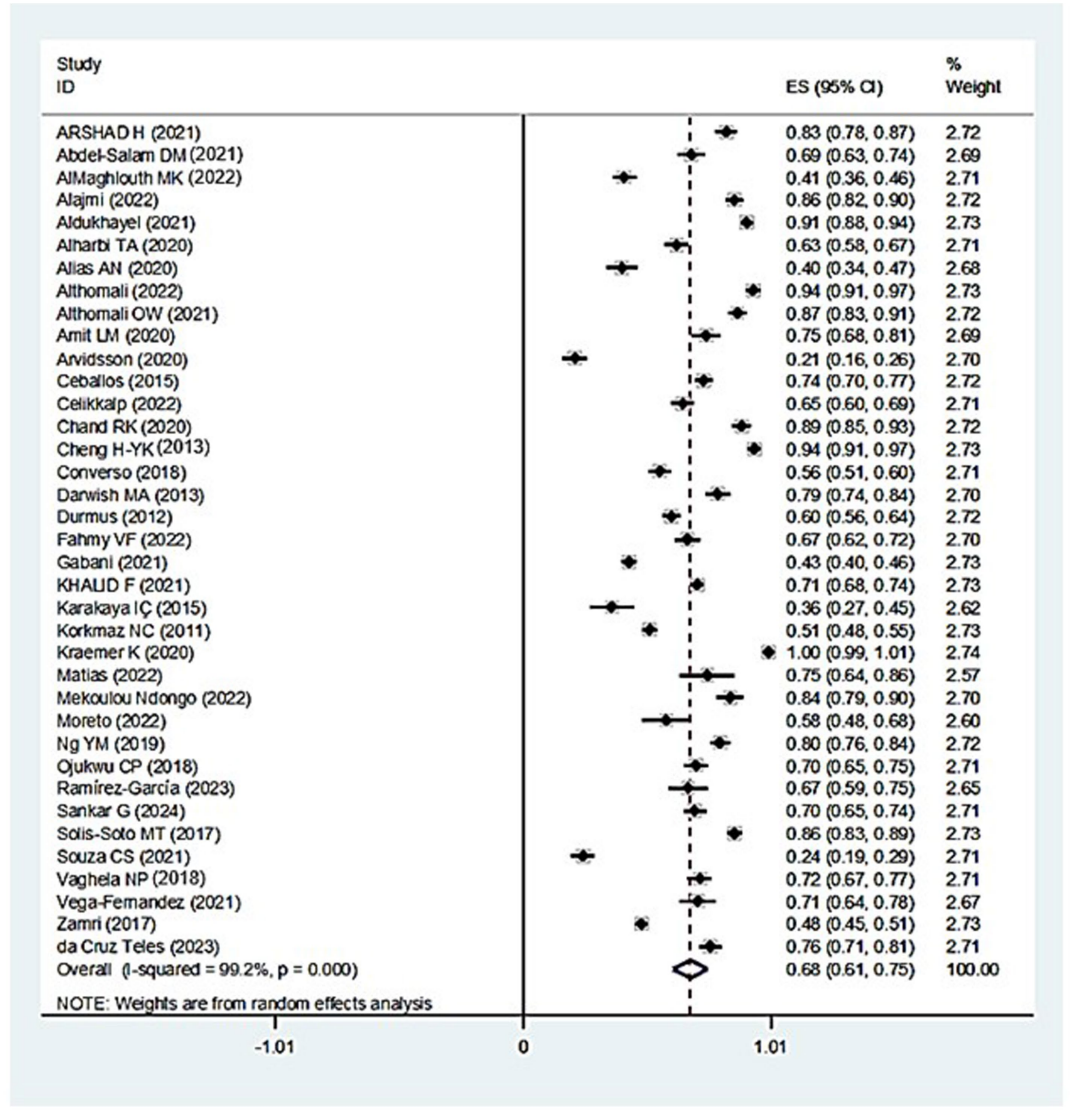
Publication bias based on Begg’s test for overall prevalence of MSDs among teachers.

Begg’s test results (*p* = 0.075) in Figure 3 reveal that there was no significant publication bias in the prevalence of overall MSDs among teachers. However, based on the outcomes of this test, the publication bias in the prevalence of MSDs in the regions of the wrist, elbow, ankle, and hip was significant.

## 4 Discussion

The present systematic review and meta-analysis aimed to examine the frequency of MSDs among teachers. After assessing 44 articles, the meta-analysis results revealed that the overall prevalence of MSDs among teachers is 68%, with the highest prevalence in the neck (47%), and lower back (47%). Furthermore, the prevalence rates of MSDs were also determined for other regions such as the shoulder (44%), upper back (37%), knee (35%), ankle (30%), wrist (27%), hip (22%), and elbow (13%).

The findings of one review study indicated that the occurrence of MSDs among general teachers varies from 48.7 to 73.7%, whereas a prevalence range of 38.7 to 94% was noted among special education teachers (8). In a separate review study by Erick and Smith focusing on the prevalence of MSDs and associated risk factors among school teachers, MSD prevalence was reported to range from 39 to 95%, with the most significant pain experienced in the upper back, neck, and upper limbs (14). The overall prevalence of MSDs in the present study was consistent with these studies. Additionally, neck pain was estimated to be the most common musculoskeletal disorder in this study and the aforementioned studies. However, although the prevalence of low back pain in the present study was estimated to be as high as neck pain, the study by Erick and Smith reported lower prevalence rates for low back pain compared to the upper back, neck, and upper limbs.

Analyzing the findings from the research previously conducted and the present study reveals that numerous teachers face the risk of MSDs as a result of being exposed to ergonomic hazards such as awkward postures and extended periods of standing (42). Prolonged standing can lead to issues such as leg swelling and pain, varicose veins, back discomfort, and neck and shoulder pain, as well as other physical ailments. Moreover, extended periods of standing reduce the blood flow to the muscles, which can hasten the onset of fatigue, resulting in muscle pain in the arms, back, and neck (43).

In various professions, the frequency of MSDs holds significant importance, and numerous studies have been carried out in this field. In a review conducted by Lietz et al., the prevalence of MSDs among dental practitioners varied from 10.8 to 97.9%, with the highest rate (58.5%) observed in the neck region (27). According to findings from another review study, the prevalence of MSDs among orthopedic surgeons was documented as 73.8% (28). In a different review study conducted among sanitary workers, the worldwide occurrence of MSDs was 40.52% (29). The comparison between the findings in the literature and the present study indicate that the prevalence rates of MSDs among teachers are higher than those among sanitary workers. However, when compared to the prevalence of these disorders among orthopedic surgeons and dentists, there was no much difference. Moreover, in most of the mentioned studies, the neck and lower back were among the most common regions affected by MSDS symptoms, which is consistent with the results of the present study.

Therefore, teaching at school is considered one of the professions with the highest global prevalence of MSDs (44). Researchers have highlighted that the job responsibilities of teachers, such as instructing students, lesson planning, grading assignments, and performing school administrative tasks, can lead to discomfort in their upper and lower limbs (9, 19). Some research has identified physical factors such as extended periods of standing and awkward writing postures as exacerbating factors for MSDs (45). Besides physical ergonomic risk factors, individual and psychosocial risk factors can also play a role in the occurrence of MSDs. For instance, factors including work experience, age, gender, body mass index (BMI), type of school, and number of students have been identified as contributors to MSDs (44). Furthermore, another study indicated that psychosocial elements such as elevated work demands, limited job control, high stress levels, job dissatisfaction, monotonous tasks, and inadequate social support are significantly linked to the prevalence of WMSDs among school teachers (1). In addition, the researchers stated that sleep disorders can also lead to MSDs (46). Regarding the teaching profession, De Souza et al. demonstrated that poor sleep quality was markedly correlated with the onset of musculoskeletal symptoms among teachers (47).

According to the results of the present study, the prevalence of MSDs among teachers, especially in the neck and lower back regions, is high. Therefore, it is necessary to consider effective coping strategies, including ergonomic risk factor assessments, periodic screening of teachers for MSDs (48), designing ergonomic interventions, and conducting necessary training courses, to reduce and control MSDs. The findings of this study can likely be useful for planning and implementing corrective measures, which could lead to more effective educational services provided by teachers and, consequently, improve the quality of the educational system. These measures can include ergonomic interventions based on teaching correct postural behavior and corrective exercises, which have been effective in reducing MSDs in many occupational groups (49).

## 5 Limitations

This study had several limitations. Initially, there was heterogeneity among the studies, possibly stemming from variations in tools, sample sizes, and cut-off points in the original studies. As another limitation of this study, it is worth noting the inability to report the incidence of MSDs by gender, as this data was not available in the original studies. Furthermore, due to the limited number of tools assessed for MSDs within subgroups, conducting instrument-based subgroup analysis was not feasible. The present study was also limited by the fact that it included only research published in English.

## 6 Conclusion

The present systematic review and meta-analysis was conducted with the aim of investigating the prevalence of MSDs among teachers. The results indicate that the overall prevalence of MSDs among teachers is 68% and the highest prevalence is related to the neck (47%), and lower back (47%). This high prevalence rate of MSDs compared to other occupational groups is noteworthy and shows that MSDs among teachers can lead to a decrease in their work ability index. Therefore, it is strongly recommended to take necessary measures to prevent MSDs, especially in the neck and back regions among teachers. It is suggested that in the future, comprehensive studies be conducted in relation to the identification of ergonomic risk factors in the teachers’ work environment and the design of ergonomic interventions for them. In addition, it is recommended that teachers be periodically screened for MSDs. Perhaps the implementation of these corrective measures will reduce the prevalence of MSDs among this occupational group.

## Data Availability

The original contributions presented in the study are included in the article/supplementary material, further inquiries can be directed to the corresponding author.
